# A Hypothetical Energy-Dissipating Mechanism Regulated by Glucose in β-Cells Preceding Sustained Insulin Secretion

**DOI:** 10.3390/cells14211644

**Published:** 2025-10-22

**Authors:** Jorge Tamarit-Rodriguez

**Affiliations:** Biochemistry Department, Medical School, Complutense University, 28040 Madrid, Spain; tamarit@ucm.es

**Keywords:** beta cell bioenergetics, connexin36, Cx36 hemichannels, glucose oxidative metabolism, insulin secretion

## Abstract

In this review we propose the hypothesis that an energy-dissipating process precedes the continuous stimulation of insulin secretion by glucose. This process is mediated by connexin 36 hemichannels (Cx36H), or Cx36 connexons. Cx36H oligomers are expressed at the plasma membrane, and their gating activity (opening) is activated by plasma membrane depolarization after the closure of K^+^ATP channels by glucose (>5 mM) metabolism. This initial depolarization (1st step) might be responsible for the first phase of insulin secretion, with the subsequent opening of Cx36H increasing β-cell plasma membrane permeability, allowing for the efflux of metabolites (less than 1KD) (GABA, adenine nucleotides) and K^+^ (2nd step). This provokes a breakdown of oxidative glucose metabolism and the repolarization of the plasma membrane. As the extracellular glucose concentration increases further (>>5 mM), it exerts a progressive inhibition effect on Cx36H opening, allowing for the continuous stimulation of insulin secretion (3d step, second phase,). The glucose feature of regulating Cx36H closing with sigmoidal kinetics (8 mM IC_50_ and around 20 mM at maximum) has been confirmed in mouse Cx36 connexin expression in Xenopus oocytes and in mouse islets stimulated by a range of glucose concentrations in the presence of 70 mM KCl. This gating activity was also inhibited by some non-metabolized glucose analogs. Glucose inhibition of Cx3H opening might not only contribute to making the insulin secretory response more specific for glucose but might also play a role in the pulsatility of sustained insulin secretion. Cx36H opening also offers the opportunity to potentiate the secretory effect in vivo by, permeant or not, metabolic stimuli. Confirmation of this novel physiological role for Cx36H in β-cells would place them as new susceptibility locus for type 1 and type 2 diabetes, whose physiological implication in the mechanism of insulin secretion regulation should be evaluated by in vivo studies in diabetic patients.

## 1. Introduction

The finding that connexin 36, specifically expressed in β-cells, is sensitive to plasma membrane depolarization, and other extracellular ionic changes, after its oligomerization to Cx36 hemichannels (Cx36H) comprises the background behind the hypothetical mechanism of bioenergetic β-cell regulation. The resultant changes in Cx36H are regulated by extracellular glucose concentration [1].

The original finding that led to the proposed hypothesis was that KCl β-cell depolarization, overlapping a metabolic stimulus, partially deranges the insulin secretory response, with 70 mM KCl significantly decreasing the second-phase response to 20 mM of glucose, either in the absence or presence of 10 mM glutamine (−35%, *p* < 0.03; −47%, *p* < 0.05) [2]. However, the islet’s pre-exposure to 5 mM glucose plus 70 mM KCl for 45 min induced a stronger suppression of the first (−69%, *p* < 0.01) and second (−49%, *p*< 0.04) phases of insulin secretion stimulation by 20 mM glucose for 30 min [2].

The simultaneous combination of 70 mM KCl together with some branched-chain oxoacids induced a very strong suppression of islet insulin secretion, as compared with control islets in the absence of KCl. As an example, 10 mM KIC (oxo-4-methylpentanoate, OMP) suppressed approximately 61% of second-phase secretion (*p* < 0.0001). Similar results were obtained with KC (oxo-3-methylbutyrate) [3].

Additionally, branched-chain oxoacids have been demonstrated to promote metabolic flux in the GABA shunt, strongly diminishing γ-amino acid content in islets [3]. KCl depolarization was also demonstrated to induce a release of intracellular GABA, cutting off the supply of substrate to the GABA shunt. Moreover, it was possible to demonstrate an increase in the islet release of GABA and taurine by more than 100% with 70 mM KCL in islets pre-loaded with 10 mM glutamine, as well as a decrease in islet content (−32 and –40%, respectively).

## 2. Experimental Data Supporting the Proposal

### 2.1. Structural Characteristics of Connexins

The mammalian family of connexins comprises 20 different members codified by different genes [4] and represented as nCxZ (n, species; Z, molecular weight). Cx36 is expressed in neurons and glia, adrenal medullary chromaffin cells, and pancreatic β-cells [5]; the latest specifically express Cx36 only, due to their absence of the transcriptional repressor, RE-1 Silencing Transcription Factor (REST) [6]. In the transition from endoplasmic reticulum to the Golgi, 6 identical or different members oligomerize to constitute a quaternary protein structure named the homomeric or heteromeric connexon, depending on the presence of a single member or a mixture of them (Cx36 connexons are homomeric). Connexons inserted in the plasma membrane constitute transmembrane channels allowing for the diffusion of ions, second messengers, and metabolites between the cytoplasm and the extracellular space, with an exclusion limit around 1 kD. They are also deemed hemichannels because they are the precursors of gap junction channels resulting from the tight apposition of two connexons of adjacent cells, allowing for molecular exchange between the two coupled cells (ionic and metabolic coupling). They cluster in lipid rafts of the plasma membrane, where calcium-dependent cell adhesion molecules promote close contact between adjacent cells (“gap junctions”).

### 2.2. Functional Features of Connexins

The physiological function of gap junction channels is better understood than the role of uncoupled connexons or hemichannels. While Cx36 gap junction channels mediate the ionic and molecular exchange between β-cells, Cx36H communicates the β-cell cytosol to the extracellular space. The group of Meda P. et al. [4] explored the role of Cx36 gap junction channels in islets and explained their role in the coordinated regulation of insulin secretion to glucose in the islet cores of β-cells [7]. However, the physiological role of hemichannels has yet to be firmly established, both in general and in β-cells in particular. An earlier study failed to detect nuclear staining with ethidium bromide on MIN6 cells or freshly isolated mouse islets incubated in 20 mM of glucose [8]. In 20 mM glucose, MIN6 cells released some ATP, which was increased by tetraethyl ammonium, even further in the absence of extracellular Ca^2+^. However, as ATP release was prevented by 200 µM of diazoxide, the authors concluded that it was possibly mediated by the exocytotic pathway. With the information presently available, it is possible to assume that the failure to detect Cx36 hemichannels was likely due to the presence of a high concentration of glucose (20 mM) in the experiments that, as explored below, would have exerted an inhibitory effect on Cx36 hemichannel gating (opening). Moreover, MIN6 cells seem to have a low expression of the mCx36 gene [9].

### 2.3. Properties of CX36H Overexpressed in Xenopus Oocytes

Connexins exogenously expressed in *Xenopus* oocytes exhibit gating properties; the molecular conductance is approximately twice that of gap junction channels. According to the authors, “the most compelling data on the properties of unpaired connexons or hemichannels was initially derived from oocytes expressing Cx46 and more recently Cx50”. In Cx43 hemichannels, conductance activates around 0 mV and increases dramatically at +60 mV; then, it decays to a residual rate, and, in the absence of divalent cations (Ca^2+^ and Mg^2+^), hemichannel opening becomes more active at negative potentials [10]. As gating regulation differs among the different connexins, it is crucial to characterize the regulatory properties of Cx36, the unique connexin expressed in β-cells.

Overexpression of the mCx36 gene (GJD2) in Xenopus oocytes increases plasma membrane permeability, as revealed by the uptake of extracellular propidium iodide under normal conditions (−42 ± 5 mV, 1.5 mM Ca^2+^, and 1 mM Mg^2+^) [1]. Electrophysiological studies showed that Cx36H gating (opening) activity was increased in the range of plasma membrane potential between–40 and +100 mV, and membrane permeability was further enhanced by omitting divalent cations from the incubation medium [1]. Cx36 hemichannel gating activity was deactivated by returning to a state of hyperpolarized potential, and voltage activation of Cx36H opening was almost completely suppressed by 10 µM mefloquine (a known Cx36 inhibitor without off-target effects in the range of 10 to 50 µM) [1,11]. More relevantly, glucose blocked hemichannel currents that were activated by depolarization in a dose-dependent (sigmoidal) manner, with an IC_50_ close to 8 mM and inhibition measuring approximately 85% in 20 mM of glucose [1].

### 2.4. ATP Movements Through Plasma Cx36H Hemichannels Activated by Plasma Membrane Depolarization in Murine Islets at Different Glucose Concentrations

#### 2.4.1. ATP Release

The gating regulation of Cx36H may vary when expressed in native β-cells. Therefore, Cx36H opening was indirectly evaluated in isolated islets with the aim of measuring the efflux of molecules, sensitive to inhibition by mefloquine. Mouse islets subject to 70 mM KCl depolarization in 5 mM of glucose showed an increased release of ATP, measured with luciferin/luciferase, which was almost completely suppressed by 20 mM glucose. Islets from the germinal knockout model of the Cx36 gene (Cx36^−/−^ mouse) did not release ATP after depolarization at any glucose concentration [1].

#### 2.4.2. Changes in β-Cell ATP Content and ATP/ADP Ratio in Rat and Murine Islets

Rat islets depolarized in 3 mM glucose in the presence of 250 µM diazoxide showed a gradual and statistically significant decrease in their ATP content as larger depolarizations were induced (15, 30, and 70 mM KCL) without any modification of their ATP/ADP ratios [1]. 15 mM KCl drove the membrane potential to the level of the plateau phase reached by high glucose (−21 mV); 40 mM of KCl depolarized the membrane by 40 mV, and the potential stabilized at the peak level of the Ca^2+^ spikes. These KCl-driven depolarizations were of a similar magnitude to those induced by glucose. The accompanying islet ATP decline was completely prevented by the presence of 20 mM glucose; medium Ca^2+^ omission did not affect ATP depletion, excluding the fact that ATP release was mediated by exocytosis. Mefloquine (a Cx36 hemichannel inhibitor) also prevented the depolarization-induced (70 mM of KCL) loss of islet ATP.

Other metabolic secretagogues, such as KIC (oxo-4-methylpentanoate (KIC) and semialdehyde succinic acid (SSA), increasing islet ATP content and the ATP/ADP ratio to the same levels as those of 20 mM glucose, failed to maintain ATP content under the same depolarizing conditions (in the presence of 70 mM KCl) [1].

The restoring effect of 20 mM glucose not only prevented the fall in islet ATP but also increased both ATP content and the ATP/ADP ratio to stimulatory values for secretion in the presence of 70 mM KCl in a sigmoidal dose-dependent manner; a half maximal effect was observed at 8 mM, and a maximum effect was observed around 20 mM, with these being remarkably similar to the sigmoidal relationship between glucose concentration and stimulated insulin secretion, suggesting its possible implication in the physiological mechanism of insulin secretion stimulation [1].

Similar results were obtained in native mouse islets depolarized with 70 mM KCl in 5 mM glucose [1]; islet ATP content was reduced approximately three-fold without changing the ATP/ADP ratio. Moreover, ATP depletion was prevented by 50 µM mefloquine and 20 mM glucose. To rule out the possibility that 20 mM glucose might counteract ATP loss via increased metabolism, the effects of non-metabolized (L-glucose, 3-0-mehyl-glucose) or poorly metabolized (D-galactose) structural glucose analogs were evaluated. Each of the analogs at 15 mM exhibited significant islet ATP content recovery without modification of the ATP/ADP ratio [1]. Glucose inhibition of KCl-induced islet ATP depletion showed a dose-dependent response, with an IC_50_ value measured at 8 mM and a maximum effect measured at 20 mM; moreover, it also increased both islet ATP and the ATP/ADP ratio, to stimulatory values for secretion in the presence of 70 mM KCl. The lack of effect of glucose on the gating of gap junction channels might be due to the occlusion of the glucose site on CX36H after the tight association of two hemichannels in the formation of a gap junction channel between neighboring β-cells. By contrast, mefloquine inhibited the conductance of both Cx36H and gap junction channels.

#### 2.4.3. Changes in β-Cell ATP Content and ATP/ADP Ratio in Transgenic Islets with Homo- and Heterozygous Knockout of Connexin 36 (Cx36^−/−^ and Cx6^+/−^ Murine Islets) [1]

The Cx36^−/−^ islets showed abnormally elevated ATP levels in 5 mM glucose, which were significantly close (no significant difference) to those reached in 20 mM glucose, albeit presenting an ATP/ADP ratio characteristic of non-stimulated islets. It is worth inquiring whether this phenomenon indicates that native islets, opposite Cx36^−/−^ islets, lose adenine nucleotides regularly to maintain a basal cytosolic ATP concentration. In our modest opinion, there is no information as to whether β-cells pay a high energy cost (oxidative phosphorylation) to synthesize adenine nucleotides, as compared with other biochemical or physiological loads.

The values for both basal and stimulated ATP content and the ATP/ADP ratio in heterozygous Cx36^+/−^islets were intermediate in comparison to the corresponding values in control and Cx36^−/−^islets. In 20 mM glucose, islets from the three different mouse phenotypes showed similar increases in their ATP content and ATP/ADP ratios above the basal values.

#### 2.4.4. Content and Release of GABA and Taurine Mediated by the Activation of Plasma Membrane Depolarization of Cx36 Hemichannels in Rat Islets [12]

Mouse islets depolarized in 5 mM of glucose by 70 mM of KCl in the presence of 250 µM of diazoxide showed a decrease in their GABA and taurine content and an increase in their release; these effects were potentiated by the omission of extracellular calcium concentration. The combination of KCl and Ca^2+^ omission resulted in a slower rate of inhibition by mefloquine, which caused a partial recovery of GABA content and release at 50 µM.

#### 2.4.5. Cell Insulin Secretion Induced by Glucose in the Absence of Cx36 [1]

A. In vivo effects

Cx6^−/−^ mice showed significantly greater glucose excursions in plasma glucose at 15 and 30 min in an intraperitoneal glucose tolerance test; the area under the curve was significantly higher than in wild-type mice. Moreover, basal plasma insulin levels were significantly lower in Cx6^−/−^ mice.

B. In vitro stimulation of insulin secretion

A gradual decrease in the insulin response to 20 mM glucose was observed in the islet phenotypes, from controls (Cx36^+/+^) to transgenic islets [1]; the Cx36^+/−^ response was intermediate between controls and Cx36^−/−^ which exhibited a single and diminished first phase. Both phases (first and second) of insulin secretion were significantly suppressed as compared with native islets; however, the first phase was always present, apart from the absence of Cx36H. Mefloquine partially diminished 20 mM glucose-induced secretion in Cx36^+/+^ and Cx6^+/−^ perifused islets.

C. Secretory effect of extracellular ATP on insulin secretion in native and transgenic mouse islets with knockout of the Cx36 gene and native rat islets.

These experiments were designed to confirm that open (by 70 mM KCl) Cx36H allowed for adenine nucleotide diffusion in two senses, dependent on the concentration gradient between the extracellular space and cytoplasm. This might also help indicate whether extracellular ATP, in 5 mM glucose, could stimulate sufficient and sustained insulin secretion. In control (Cx36^+/+^) islets, only the first phase of insulin secretion (due to KCl) was not modified by 5 mM ATP, but it induced a significant second phase (*p* < 0.05). Cx36^+/−^ islets responded to 5 mM ATP with a weak stimulation of the first and second phases of secretion; no secretory response was recorded in Cx36^−/−^ islets.

## 3. Bioenergetic Regulation of β-Cells

A review on the regulation of β-cell bioenergetics, written by Prof. Nicholls D.G., brilliantly describes the general concepts necessary to understand it [12]. Therefore, a paragraph of the review has been translated, included below:

“In the β-cell, the downstream dissipative pathways that balance the upstream delivery of glucose-derived pyruvate to the mitochondrion are of critical importance but are commonly neglected. These include the abnormally high endogenous mitochondrial proton leak and the largely unexplored quantitation of ATP utilizing pathways. In the absence of these dissipative pathways, even the slowest trickle through the glycolytic pathway would eventually result in a maximal ATP/ADP ratio approaching thermodynamic equilibrium. It is therefore important to understand how the β-cell, which has evolved to vary the Gibbs free energy of the cytosolic ATP hydrolysis reaction (“ΔGp = ΔG_ATP_”) as a function of upstream substrate availability, differs from, for example, heart muscle, which possesses feedback mechanisms to ensure that ΔGp (“mitochondrial electrochemical membrane potential or proton motif force”) remains as constant as possible in response to huge variations in ATP demand”. In our opinion, the nature of the dissipating pathway implicated in the balanced regulation of mitochondrial oxidative phosphorylation in β-cells has not yet been defined.

### 3.1. Does Cx36 Hemichannel-Dependent Repolarization Oppose K^+^ATP Channel-Dependent Depolarization?

**Figure 1:**
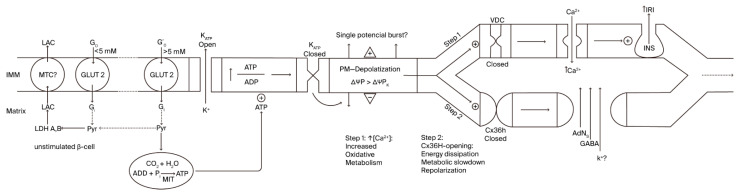
Relationship between variations in β-cell plasma membrane voltage and bioenergetic mitochondrial function.

As demonstrated earlier, at basal glucose levels (0 to 3–5 mM), β-cell glucose metabolism is essentially anaerobic, and both ATP production and the cytosolic ATP/ADP ratio must be low [13]. Above 5 mM, glycolytic-derived pyruvate begins to oxidize in mitochondria [14], and the ATP generated in oxidative phosphorylation closes K^+^_ATP_ channels, causing plasma membrane depolarization (first step; Figure 1). This initial step activates voltage-dependent Ca^2+^ channels, and the resulting increase in cytosolic Ca^2+^ activates mitochondrial metabolism, which then activates insulin secretion. It is tempting to speculate as to whether a burst of membrane potential depolarization is responsible for the first phase of insulin secretion in the first step of β-cell activation, as observed in pioneering recordings depicting short insulin secretion stimulation by glucose, followed by a period of electrical silencing [14,15]. Perhaps more physiological Ramp stimulations involving glucose would lead to better intermingling of both processes.

According to our hypothesis, membrane depolarization activates the gating of Cx36 hemichannels (unknown timing relationship) and facilitates the communication between cytosol and extracellular milieu (second step; Figure 1). This means that β-cell membrane permeability is increased, not only facilitating the exchange of molecular components but perhaps also contributing to antagonizing membrane depolarization due to a certain dissipation of the K^+^ gradient [10]. Whether the repolarization current is of a similar magnitude, or one that is small enough, to not compete with the K^+^_ATP_-dependent depolarization current has yet to be established. It must depend on the number of Cx36 hemichannels compared to the K^+^_ATP_ channels.

### 3.2. Cx36 Hemichannels Mediate the Release of Important Molecular Entities Necessary for an Intact Mitochondrial Metabolism (ATP and GABA)

Cx36 hemichannel opening allows for the extracellular efflux of important molecular components needed to mediate the stimulus–secretion coupling mechanism of glucose-induced insulin release. The loss of islet intracellular GABA, highly implicated in the coupling mechanism between the citric acid cycle and the GABA shunt, as demonstrated by several metabolic stimuli (glucose, branch-chain ketoacids, semialdehyde succinic acid), restricts the oxidative metabolism of said stimuli [16]. Due to the limiting activity of β-cell lactate dehydrogenase, no further increase in anaerobic glycolysis is possible (absence of the Pasteur effect) to improve substrate-level ATP synthesis. Moreover, the loss of cytosolic adenine nucleotides, ADP, might maintain mitochondrial respiration at state IV, interrupting oxidative phosphorylation. The suppression of the cytosolic adenine nucleotide pool might also reduce the Gibbs energy released in the hydrolysis of ATP (↓ ΔG_ATP_), also contributing to metabolic slowdown (metabolism stop due to the suppression of the cytosolic ATP/ADP ratio). The accompanying fall of the electrochemical mitochondrial membrane potential (↓ΔGp) would also, perhaps primordially, condition the value of cytoplasmic ΔG_ATP_, which would likely also contribute to the slowdown of β-cell glucose metabolism by deficient substrate phosphorylation [12].

First, to the left, the plasma membrane of a β-cell at rest (glucose concentration < 5 mM), characterized by a zero net flux of K^+^ at its K^+^-equilibrium potential (ΔΨP_K_ = −60 mV). This resting β-cell predominantly depends predominantly on anaerobic glycolysis, with a restricted ATP production rate due to the absence of mitochondrial oxidative metabolism (OxPhos). As extracellular glucose concentration rises (>5mM), glycolysis exceeds the capacity of lactate dehydrogenase (LDH) to generate lactate (LAC), and the extra pyruvate (Pyr) produced is delivered to the mitochondria for its oxidative metabolism, increasing cytosolic ATP concentration (Step 1). Subsequently, K^+^_ATP_ channels are closed, and the plasma membrane depolarizes (ΔΨP > ΔΨP_K_; less negative), exhibiting an oscillatory burst of action potentials caused by the increased influx of Ca^2+^, due to the opening of the voltage-dependent channels (VDCs). The final consequence effect is the stimulation of insulin secretion (first phase?). In Step 2, the previous membrane depolarization induces repolarization and the opening of Cx36 hemichannels, resulting in the repolarization of the plasma membrane, due to a glucose metabolism slowdown and an increased efflux of K^+^. MCT (plasma membrane monocarboxylate transporter); GLUT 2 (glucose transporter 2).

### 3.3. Proposed Mechanism of Bioenergetic Regulation in β-Cells

#### 3.3.1. Antecedents

**Figure 2:**
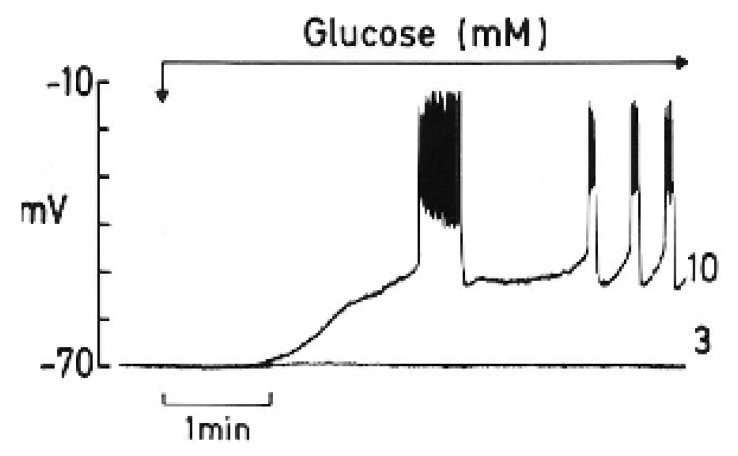
The effect of a change in glucose concentration from 3 to 10 mM on the membrane potential of a single mouse β-cell (graphic kindly provided by Henquin J.C.).

To appreciate the parallelism observed between the two processes, a summary of a review by Henquin J.C. [15] is worth highlighting, as follows: “The timing of the main ionic events accompanying membrane depolarization by glucose are as follows (see Figure 2): 1. A square-wave increase in glucose concentration (from 3 to 10 mM) triggers initially an increase in Rb^+^ (tracer for K^+^) efflux coinciding with a long first burst of membrane voltage depolarization with continuous spike activity. 2. It follows a decrease in Rb^+^ efflux, and a repolarization (silent interval) first demonstrated by Sehlin J. and Freinkel N.” [17]. According to Henquin JC [15], “it was tempting to suggest that a biphasic change in K^+^ membrane permeability contributes to the biphasic pattern of electrical activity and eventually of insulin release, but such a proposal awaits experimental support”. In other words, the first long voltage burst would be responsible for the first phase of glucose-induced insulin secretion. 3. “After the silent interval, β-cell plasma membrane depolarizes again to the threshold potential when glucose rises to 6–7 mM and repetitive slow waves are generated. This second and steady round of depolarization is probably responsible for the second phase of glucose-induced insulin secretion. A further increase in the glucose concentration does not affect the absolute value of the membrane potential, but the slow waves lengthen whereas the intervals among them shorten. At 18 mM glucose, the membrane is permanently depolarized at the plateau potential, with continuous spike activity”.

#### 3.3.2. Hypothetical Participation of the Proposed Energy-Dissipating Pathway in the Bioenergetic Regulation of β-Cells

A new proposal for the participation of Cx36H in the bioenergetic regulation of stimulus–secretion coupling provides experimental support for the implication of Cx36H in the biphasic changes observed in Rb^+^ efflux and in plasma membrane depolarization/repolarization after glucose metabolism-dependent depolarization by the closure of K^+^ channels.

**Figure 3:**
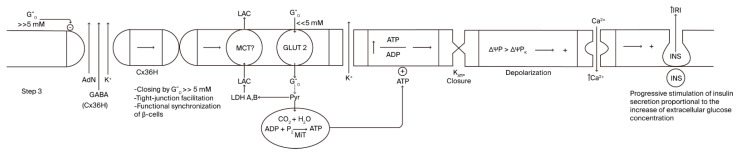
Step 3 of the stimulus–-secretion coupling mechanism of glucose- induced insulin secretion implicating the hypothetical role of β-cell-plasma membrane Cx36 hemichannels.

Cx36 hemichannel (the second step) mediates an energy-dissipative pathway just before the definitive activation of sustained, glucose-induced insulin secretion. A continuous rise in extracellular glucose concentration after a meal will lead glucose concentration to enter its inhibitory concentration range of Cx36 hemichannels, provoking their closure (third step; Figure 3). A striking finding was that the inhibition kinetics of glucose are sigmoidal, with a half concentration in 8 mM of glucose, which is significantly similar to the kinetics of glucose stimulation of insulin secretion [1]. Closing Cx36 hemichannels might favor the efflux of molecules and ions through tight junction channels, promoting the synchrony of the insulin secretory response of more β-cells in the islet core. Of course, whether a recognition site for glucose exists in the amino acid sequence or in the corresponding genetic sequence of Cx36 must be confirmed.

As the extracellular glucose concentration continues to increase (>>5 mM), it enters a range of hyperbolic Cx36 hemi-channel inhibition, which leads to the progressive closing of the hemichannels, dependent on the final glucose concentration reached. This makes possible a partial or maximum recovery of Step 1 and all its components: K^+^_ATP_-closure by a recovered glucose metabolism, membrane depolarization, and an increase in cytosolic Ca^2+^ concentration that allows for the continuous stimulation of insulin secretion (2nd second phase?). The insulin response will return spontaneously to basal values when the extracellular glucose declines back to its basal concentration (5 mM): the corresponding suppression of glucose metabolism will proportionally decrease OxPhos, the cytosolic ATP/ADP ratio, and the stimulation of insulin secretion.

## 4. Two Different Ways of Regulating a Steady Cellular Bioenergetics Transition

According to various authors, many cells accelerate their oxidative phosphorylation processes when they experience a physiological workload with an important ATP cost that is balanced by an increased OxPhos. These cells are categorized as “fuel utilizers”, and they are activated by “ATP demand”. Moreover, their cytosolic ATP levels are constant, independent from their stimulation [18,19].

Others, such as β-cells, react to a stimulus increasing their cytosolic ATP content, which is dependent on the stimulus concentration (glucose in the β-cells) and not on an increased physiological workload. These cells are considered “fuel sensors”, and their oxidative phosphorylation is hypothesized to be regulated by “ATP supply” but not by “ATP demand” [18]. However, this concept is now debated.

At variance with fuel utilizer cells, stimulated β-cells increase their OxPhos before there is any ATP demand (increased insulin secretion), simply by an increase in extracellular glucose concentration and its subsequent oxidative metabolism. This is accompanied by an elevation of cytosolic ATP and the ATP/ADP ratio, in contrast to that in utilizer cells. OxPhos and all its effects return to basal levels when the extracellular glucose concentration returns to its basal level.

In the case of β-cells, an energy-dissipative event (Step 2) seems to precede the sustained (Step 3) stimulation of insulin secretion mediated by the opening of Cx36Hs after the initial depolarization of the plasma membrane by K^+^_ATP_ channel closure (Step 1). This is responsible for transient repolarization after K^+^_ATP_ channel closure and resetting oxidative phosphorylation to a minimum value that might increase the stimulation span of subsequent glucose stimulation. Moreover, this prevents any increased stimulation of oxidative metabolism by any other non-glucose metabolites. More importantly, the subsequent transition to sustained stimulation (third phase) is left under the specific control of the conditions set by the rise in extracellular glucose concentration.

## 5. Experimental Conditions Considered to Be Indicative of ATP Demand in β-Cells

The energy consumption of the main ATP-demanding cellular processes and their dependence on OxPhos have been measured in some mammalian cells (categorized as “fuel utilizers”), with results uncovering a bioenergetic hierarchy in which higher O_2_ (ATP) consumers were protein and RNA/DNA synthesis, followed by sodium cycling through the Na+/K+-ATPase, the Ca+ ATPase, unidentified ATP consumers, and proton leaks [20].

There is virtually general agreement that sulfonylureas, in general, suppress islet ATP levels and that this is due to an excess of ATP consumption over its synthesis provoked by an excessive workload, likely due to a high stimulation of glucose-induced insulin secretion (?). However, glucose-induced secretion is also generally reduced by the combination of glucose and sulfonylureas: Tolbutamide [21,22]; Glibenclamide [23] and Gliburide [24]. In support of this, Detimary P. et al. [22] demonstrated the finding that 30 mM of KCL significantly suppressed the ATP/ADP ratio of mouse islets incubated in 10 mM of glucose.

Stimulation of the plasma membrane Na^+^-K^+^-ATPase with glibenclamide and meglitinide, as opposed to depolarized β-cells, significantly suppressed islet concentrations of all three adenine nucleotides in 10 mM glucose without affecting glucose oxidation. The effect was also attributed to elevated ATP consumption by an activated Na^+^-K^+^-pump [25].

All these experimental maneuvers intended to increase the ATP demand of β-cells unexpectedly resulted in their exhaustion, likely caused by a loss of clue metabolites (GABA; adenine nucleotides) because of an excessive depolarization-induced opening of β-cell hemichannels.

Although the energetic cost of insulin exocytosis has not been measured in β-cells (see reference [20] to know the procedure to do it, mentioned in the first paragraph of this section) but in “fuel utilizers” cells, an intuition of its magnitude may be obtained from some experimental data: (1) From Figure 2 one can calculate that the time between the first burst of β-cell plasma membrane potential (1st phase of secretion?) and the following continuous recording (2nd phase of secretion) is around 1 min. This silent interval (1 min) is, according to our proposal, due to the opening of Cx36 hemichannels. (2) Pre-exposure of perifused rat islets to 70 mM KCl at 5 mM glucose for 45 min, suppressed both phases of secretion induced by 20 mM glucose by 69 (1st phase) and 49% (2nd phase), respectively [2]. However, the suppression of the second phase of secretion triggered by 20 mM glucose plus 70 mM glucose, without previous exposure to KCl (was smaller (35%) [2]. (3) The second phase of secretion triggered by 10 mM KIC (oxo-4-methylpentanoate) in the presence of 70 mM KCl was suppressed within 61% after 30 min [3]. It may be concluded that the loss of molecular components required for insulin secretion stimulation needs several minutes to produce an inhibition of secretion. This conclusion also suggests that as the extracellular glucose concentration increases, the duration of Cx36 opening shorts and so does the silent phase between bursts during sustained secretion, in proportion to the glucose concentration.

## 6. Cx36 Gating Opens a Window of Opportunity to Potentiate Glucose-Induced Secretion by Glucose

This aspect of the stimulus–secretion coupling process of insulin secretion will be explained with reference to a particular set of experiments performed in rat perifused islets stimulated with glucose and succinic acid dimethyl ester (SAD), whose secretory response was strongly potentiated by pyruvate [26]. The latter does not permeate the plasma membrane of rat β-cells because of their monocarboxylate transporter deficiency (MCT1 and 2) in their plasma membrane [27].

As expected, 10 mM of pyruvate alone failed to trigger any insulin secretory response. However, it potentiated the individual responses to 20 mM of glucose and 10 mM of SAD that were of similar magnitude by +64% each [26]. Moreover, the combination of 20 mM glucose together with 10 mM SAD [26] allowed for the reproduction of potentiation by 10 mM pyruvate in the individual secretory responses to 20 mM glucose and 10 mM SAD, increasing insulin release by 60–70%. This potentiation of glucose-induced insulin secretion by pyruvate and SAD is an example of regulation by ATP supply because the resulting increased secretion load did not cause any feedback suppression.

Interpreting these experimental results, in accordance with our proposal of the bioenergetic regulation of β-cells, it is possible to conclude that the facilitated plasma membrane permeability of pyruvate might be attributed to the opening of Cx36H, occurring after plasma membrane depolarization by the closure of K^+^_ATP_ channels. This possibility would enable the potentiation of glucose-induced insulin secretion in vivo, administering metabolic stimuli, permeable or not, that would be taken up by open Cx36 connexons during Step 2 of the β-cell bioenergetic pathway. Perhaps this feature of the β-cell plasma membrane would challenge any pharmacological treatment of type 1 and/or 2 diabetes in the future.

## 7. Are Cx36 Proteins Risk Factors for the Development of Type 1 and Type 2 Diabetes?

An early report by Allagnant et al. [28] showed that 20 mM glucose repressed Cx36 gene expression in insulin-secreting cells, diminishing both its protein and mRNA levels. Glucose-induced transcription repression was ascribed to a putative cAMP response element (CRE) located between bases –566 and –556 of the Cx36 gene promoter. The D-glucose effect was not reproduced by the non-metabolized glucose analogs L-glucose and 3-*O*-methyl-D-glucose at 20 mM; however, 2-deoxy-D-glucose, phosphorylated by glucokinase, but poorly metabolized further in glycolysis or the pentose phosphate pathway, induced a 50% suppression of Cx36 protein levels. The authors did not find any glucose-responsive element (GlRE) or carbohydrate-responsive element (ChoRE) in their bioinformatic search of the human 3945 bp fragment in the human Cx36 promoter used in the experiments. It would be interesting to investigate the hypothetical Cx36 amino acid sequence, recognizing the glucose molecule and its corresponding nucleotide-codifying region in order to search for its polymorphisms in the human sequence. As our proposal is the first attributing a physiological role to Cx36 hemichannels in β-cells, it is yet unknown whether their genetic polymorphisms might affect their modulation by glucose. One must bear in mind the notion that the hypothetical Cx36 H site for glucose is not expected to affect gap junction channel gating that is supposed to be occluded by the formation of connexons.

Islet Cx36 has been implicated in both type 1 and type 2 diabetes, but its mechanisms are yet unknown. Farnsworth N.L. and Benninger R.K.P. have reviewed the putative roles of Cx36 in the development of both type 1 and type 2 diabetes [29]. Recent studies suggest that, in type 1 diabetes development, Cx36 gap junction channels modulate ER and oxidative stress, as well as apoptosis induced by proinflammatory cytokines. According to the authors, gene coding for Cx36 is a susceptibility locus for type 2 diabetes, although no direct implication of gap junction coupling has been found in humans with type 2 diabetes. Nevertheless, prediabetic mice subject to a high-fat diet experience a decrease in their Cx36 gap junction coupling, under prevailing hyperglycemic and hyperlipidemic conditions, which leads to a loss of electrical activity synchronization and [Ca^2+^]_i_ oscillations [29].

## Data Availability

No new data were created or analyzed in this review.
